# A Vegetal Biopolymer-Based Biostimulant Promoted Root Growth in Melon While Triggering Brassinosteroids and Stress-Related Compounds

**DOI:** 10.3389/fpls.2018.00472

**Published:** 2018-04-10

**Authors:** Luigi Lucini, Youssef Rouphael, Mariateresa Cardarelli, Paolo Bonini, Claudio Baffi, Giuseppe Colla

**Affiliations:** ^1^Department for Sustainable Food Process, Università Cattolica del Sacro Cuore, Piacenza, Italy; ^2^Department of Agricultural Sciences, University of Naples Federico II, Portici, Italy; ^3^Consiglio per la Ricerca in Agricoltura e l’Analisi dell’Economia Agraria, Centro di Ricerca Orticoltura e Florovivaismo, Pontecagnano Faiano, Italy; ^4^NGA Laboratory, Tarragona, Spain; ^5^Department of Agricultural and Forestry Sciences, University of Tuscia, Viterbo, Italy

**Keywords:** biostimulants, *Cucumis melo* L., hormone-like activity, lignosulfonates, metabolomics, peptides

## Abstract

Plant biostimulants are receiving great interest for boosting root growth during the first phenological stages of vegetable crops. The present study aimed at elucidating the morphological, physiological, and metabolomic changes occurring in greenhouse melon treated with the biopolymer-based biostimulant Quik-link, containing lateral root promoting peptides, and lignosulphonates. The vegetal-based biopolymer was applied at five rates (0, 0.06, 0.12, 0.24, or 0.48 mL plant^-1^) as substrate drench. The application of biopolymer-based biostimulant at 0.12 and 0.24 mL plant^-1^ enhanced dry weight of melon leaves and total biomass by 30.5 and 27.7%, respectively, compared to biopolymer applications at 0.06 mL plant^-1^ and untreated plants. The root dry biomass, total root length, and surface in biostimulant-treated plants were significantly higher at 0.24 mL plant^-1^ and to a lesser extent at 0.12 and 0.48 mL plant^-1^, in comparison to 0.06 mL plant^-1^ and untreated melon plants. A convoluted biochemical response to the biostimulant treatment was highlighted through UHPLC/QTOF-MS metabolomics, in which brassinosteroids and their interaction with other hormones appeared to play a pivotal role. Root metabolic profile was more markedly altered than leaves, following application of the biopolymer-based biostimulant. Brassinosteroids triggered in roots could have been involved in changes of root development observed after biostimulant application. These hormones, once transported to shoots, could have caused an hormonal imbalance. Indeed, the involvement of abscisic acid, cytokinins, and gibberellin related compounds was observed in leaves following root application of the biopolymer-based biostimulant. Nonetheless, the treatment triggered an accumulation of several metabolites involved in defense mechanisms against biotic and abiotic stresses, such as flavonoids, carotenoids, and glucosinolates, thus potentially improving resistance toward plant stresses.

## Introduction

A well-developed root system is an important agronomic trait of horticultural crops, with implications on crop productivity, abiotic stress tolerance as well as nutrient uptake and assimilation (Koevoets et al., 2016). In fruiting vegetables (i.e., tomato, eggplant, pepper, melon, and watermelon), the most intense root growth development is usually concentrated during the first phenological stages and decreases over time due to the limited translocation of photosynthates (i.e., soluble sugars) resulting from the strong sink demand for reproductive growth (Heuvelink, 2005). Therefore, a vigorous and well-developed root system during early phenological stages able to explore the soil volume is a primary requisite for securing yield stability under both optimal and suboptimal conditions (Jung and McCouch, 2013).

Breeding and genetic engineering efforts to improve crop productivity are practically focused on shoot traits, whereas the root traits are still not fully exploited source of crop improvement (Wachsman et al., 2015; Koevoets et al., 2016). More recently, a variety of biostimulant substances (i.e., humic and fulvic acids, protein hydrolysates, and seaweed extracts) and microbial inoculants (i.e., mycorrhizal fungi and plant growth promoting rhizobacteria-PGPR) has been introduced as an efficient, safe, and sustainable tool to optimize root system thus boosting crop performance, and nutrient use efficiency as well as enhancing tolerance to environmental stressors (du Jardin, 2015; Colla et al., 2015a, 2017a; Rouphael et al., 2015, 2017b,c). The stimulation of biomass production in response to biostimulant application has been usually associated to the action of specific *signaling molecules* on plant metabolism and physiology (Colla et al., 2015b, 2017a).

Over the past decade, vegetal-based biopolymers like peptides and lignosulphonates have gained prominence worldwide as biostimulant molecules in vegetable cropping systems (Ertani et al., 2011; Matsumiya and Kubo, 2011; Colla et al., 2013, 2014, 2017b). Matsumiya and Kubo (2011) have shown that bioactive peptides isolated from soybean seeds had phytohormone-like activities since they able to promote the root hair formation (root hair length and numbers) of annual baby’s-breath, cabbage, Italian clover, and lettuce. Furthermore, Colla et al. (2014) also demonstrated a root stimulation effect induced by a commercial legume seeds-derived protein hydrolysate Trainer. The mode of action through which Trainer improves the absorption and transport of nutrients may be at least partially associated to changes in root growth with lateral root formation through an auxin-signaling mediated pathway (Colla et al., 2014, 2015a, 2017a). In addition, also lignosulphonates have been shown to elicit both auxin and gibberellin-like activities incurring a significant increase in maize biomass (Ertani et al., 2011). In their study Ertani et al. (2011) demonstrated that lignosulphonates treatment improved N assimilation in maize plants through the stimulation of key enzymes (glutamate-synthase and glutamine-synthetase) and also by promoting photosynthetic activity through the stimulation of both rubisco enzyme activity as well as chlorophyll biosynthesis. Besides the biostimulant-mediated enhancement of root growth, vegetal-based biopolymers can also upregulate the expression of genes encoding for plasma membrane H^+^-ATPases and nitrate transporters, resulting in greater efficiency in nitrate uptake and assimilation (Tavares et al., 2017).

Despite the above described effects of biopolymers in plants, conclusive evidences regarding their molecular targets are far from being elucidated. In this perspective, untargeted profiling approaches by metabolomics have proven to be a powerful tool for shedding light on the mode of action of vegetal-based biopolymers (Lucini et al., 2015, 2016; Ntatsi et al., 2017). Indeed, metabolomics allows identifying those compounds altered by treatment, thus driving optimal target uses of biopolymers-based products in agriculture. In turn, this information on the mechanisms through which biostimulants act in plant, can drive a better use of these products by highlighting those scenarios where they provide a real and essential contribution to crop production.

The elucidation of fundamental plant physiological and biochemical responses to vegetal-based biopolymer application can be instrumental to understand the mechanisms behind the well-developed root system; hence the objective of the current study was to assess the morphological, physiological, and metabolomic changes in response to vegetal-based biopolymer application on greenhouse melon. Melon plants treated with vegetal-based biopolymer by substrate drench were compared to untreated plants in terms of biomass production and partitioning, root morphology, SPAD index, chlorophyll fluorescence as well as metabolic profiling.

## Materials and Methods

### Plant Material, Growth Conditions, Biostimulant Treatments, and Experimental Design

The greenhouse trial was carried out in the spring 2016 growing season in a 300 m^2^ polyethylene greenhouse at the Experimental Farm of Tuscia University, central Italy (latitude 42°25′N, longitude 12°08′E, altitude 310 m). Transplants of melon (*Cucumis melo* L. - cv. Giorillo commercialized by Seminis, Milan, Italy) grown in polystyrene plug trays (84 holes) were planted at the second true-leaf stage on 4th May in black plastic pots (15 cm diameter; one plant per pot) containing 1.8 L of sandy soil. Fertilization was performed prior planting by mixing for each kilogram of soil 2 g of a slow mineral fertilizer containing (g kg^-1^) 150 N, 39.2 P, 124.5 K, 12.1 Mg, 80.0 S, 3.0 Fe, 0.1 B, 0.1 Mn, 0.02 Cu, and 0.02 Zn. Plants were grown under natural light conditions. The greenhouse was maintained at daily temperatures between 18 and 28°C, and day/night relative humidity of 60/85%. Just after transplanting, plants were irrigated with 200 mL of water per pot.

The five biostimulant application treatments were four rates of the commercial Quik-Link product: 0, 0.06, 0.12, 0.24, or 0.48 mL plant^-1^ (= 0, 0.3, 0.6, 1.2, or 2.4 L ha^-1^, respectively; the rates per hectare were calculated considering a localized biostimulant placement near the transplants with a plant density of 5,000 plants ha^-1^). Quik-link is a biopolymer-based biostimulant manufactured by Italpollina S.p.A., Rivoli Veronese, Italy; the product contains Lateral root promoting peptides (LRPP) and other biopolymers (e.g., lignosulphonates) with high biological activity on plants, and micronutrients (10 g kg^-1^ Fe, 7 g kg^-1^ Mn, 3 g kg^-1^ Zn, 1 g kg^-1^ Cu, 0.2 g kg^-1^ Mo). Quik-link is allowed in organic agriculture according to the Council Regulation (EC) No. 834/2007 of 28 June 2007.

Treatments were arranged in a randomized block design with four replicates. Each experimental unit consisted of 10 plants. Fifty milliliters of a water solution containing Quik-link product was applied manually after 2 days from transplanting around the collar level of each plant. During the experiment, plants were irrigated as needed with a drip irrigation system.

### Growth Measurements and Root Characteristics

At the end of the greenhouse experiment (16th May; 12 days after transplanting-DAT) melon plants were separated into leaves, stems, and roots. All plant tissues were dried at 80°C for 72 h until they reached a constant weight which corresponded to their dry biomasses. Shoot dry weight was equal to the sum of the aerial vegetative parts (leaves + stems), and the root-to-shoot ratio was also calculated. Two plants per experimental plot were used for the root morphology determination. Root system collection and sample preparation were performed following the protocol described previously by Rouphael et al. (2017a). Briefly, the melon root were gently washed with fresh water, until the roots were free from any sandy particles. The determination of the root system architecture components was done using a WinRHIZO Pro (Regent Instruments Inc., Canada), connected to a STD4800 scanner. The following root morphology characteristics were recorded: total root length, mean root diameter, and total root surface area (Colla et al., 2014).

### Soil Plant Analysis Development Index and Chlorophyll Fluorescence

Soil Plant Analysis Development (SPAD) index and the fluorescence measurements of melon leaves were also recorded at the end of the experiment. The relative leaf chlorophyll concentration expressed as a rational unit was measured using a portable chlorophyll meter SPAD-502 (Minolta Corporation, Ltd., Osaka, Japan). The SPAD measurements were made on the fully expanded leaves which correspond to the third leaf starting from the apical shoot, as described by Colla et al. (2011). Twenty random readings per experimental unit were taken and averaged to a single SPAD value for each biostimulant application treatment.

On the same date, the chlorophyll fluorescence was measured every 20 min on dark-adapted leaves (two measurements per plant) by means of a chlorophyll fluorometer Handy PEA (Hansatech Instruments Ltd., King’s Lynn, United Kingdom) with excitation source intensity higher than 3,000 μmol m^-2^ s^-1^ at the sample surface as described previously by Borgognone et al. (2016). Briefly, the minimal fluorescence intensity (*F*_0_) in a dark-adapted state was measured in the presence of a background weak light signal (about 2–3 μmol photons m^-2^ s^-1^). The maximal fluorescence level in the dark-adapted state (*F*_m_) was induced by 0.8 s saturating light pulse (3,000 μmol photons m^-2^ s^-1^). The maximum quantum yield of open photosystem II (PSII) (*F*_v_/*F*_m_) was calculated as (*F*_m_–*F*_0_)/*F*_m_, as also described by Borgognone et al. (2016).

### Collection of Samples and Metabolomic Analysis

The first fully expanded leaf (the second leaf from the growing tip) and root (terminal roots) samples were collected from two plants per experimental plot treated with 0 or 0.24 mL plant^-1^ of the biostimulant Quik-link. Leaf and root tissues were then quenched in liquid nitrogen and grounded into a fine powder using mortar and pestle, thereafter leaf and root samples were stored at -80°C for successive metabolomic analysis.

Samples (1.0 g) of five replicates per treatment were extracted by homogenization in 15 mL of 80% methanol added with 0.1% HCOOH, using an Ultra-Turrax (Ika T-25, Staufen, Germany). Extracts were centrifuged (9000 × *g*), filtered through a 0.22 μm cellulose syringe filter and then transferred to an amber vial for analysis. Metabolomic analysis was done using a 1290 UHPLC liquid chromatography system coupled to a G6550 quadrupole-time-of-flight mass spectrometer (UHPLC-ESI/QTOF-MS – Agilent Technologies, Santa Clara, CA, United States). The mass spectrometer was equipped with a JetStream dual Electrospray ionization source.

Instrumental conditions were taken from previous experiments (Pretali et al., 2016). Briefly, UHPLC separation was achieved on an Agilent Zorbax Eclipse-plus column (75 mm × 2.1 mm i.d., 1.8 μm) with a mobile phase consisting of water (A) and methanol (B), and a flow of 220 μL min^-1^ at 35°C. The gradient was operated from 5 to 90% B in 35 min, whereas the QTOF mass spectrometer was set in positive polarity, scan acquisition (100–1200 m/z^+^) and extended dynamic range mode. Nebulizer pressure was 60 psig, sheath gas was nitrogen at 10 L min^-1^ (350°C), drying gas was nitrogen at 10 L min^-1^ (280°C) and capillary voltage was 4 kV.

Features deconvolution, as well as the following mass and retention time alignment, were done in Profinder B.05 (from Agilent Technologies). Annotation was achieved using accurate mass, isotope accurate spacing and isotope ratio against the database PlantCyc 9.5 (Plant Metabolic Network^1^; released November 2014). Hence, a Level 2 of identification (i.e., putatively annotated compounds) was achieved, with reference to COSMOS Metabolomics Standards Initiative^2^. Compounds were finally filtered by frequency, retaining only those compounds that were present in 100% of replications within at least one treatment, then exported for statistics.

### Statistical Analysis of Experimental Data

Analysis of variance (ANOVA) of the experimental data was made using the SPSS software package (IBM SPSS Statistics version 20.0.0). Orthogonal contrasts (Gomez and Gomez, 1983) were used to compare the biostimulant concentration effects on morphological and physiological parameters. Duncan test was also performed at *P* = 0.05 on each of the significant variables measured.

Regarding metabolomics, the dataset was interpreted in Agilent Mass Profiler Professional B.12.06 (from Agilent Technologies) as previously reported (Lucini et al., 2017). Compounds abundance was normalized at the 75th percentile and baselined to the median of control following the adoption of a threshold of 10000 counts. Pairwise comparisons were done in Volcano Plot analysis, by combining analysis of variance (*P* < 0.05, Bonferroni multiple testing correction) and fold-change analysis (cut-off = 5). The dataset was next exported into SIMCA 13 (Umetrics, Malmo, Sweden), pareto-scaled and elaborated for Principal Component Analysis. Thereafter, Orthogonal Projections to Latent Structures Discriminant Analysis (OPLS-DA) modeling was carried out for leaves and roots, separately. OPLS-DA supervised multivariate analysis targeted separating variation between the groups into predictive and orthogonal (i.e., ascribable to technical and biological variation) components. Outliers were excluded according to Hotelling’s T2 and, adopting 95 and 99% confidence limits for suspect and strong outliers, respectively. The OPLS-DA model was validated through cross validation CV-ANOVA (*p* < 0.01) and overfitting was excluded by permutation testing (*n* = 100). OPLS-DA goodness-of-fit R^2^Y and goodness-of-prediction Q^2^Y were recorded and finally variables importance in projection (VIP analysis) was used to select those having the highest discrimination potential (VIP score > 1.46).

## Results

### Morphological and Physiological Parameters

In the current study, no significant differences on dry weight of stem, SPAD index and the maximum quantum use efficiency of PSII in dark-adapted state (*F*_v_/*F*_m_) were observed between biostimulant application rates. The mean dry weight of stem, SPAD index and *F*_v_/*F*_m_ were 32.9 mg plant^-1^, 51.2 and 0.82, respectively (**Table 1**).

**Table 1:** Effect of biopolymer-based biostimulant applications on dry weight of leaves, stems, total biomass, SPAD index, and maximum quantum use efficiency of photosystem II in dark-adapted state (*F*_v_/*F*_m_) of melon plants grown under greenhouse conditions.

Biostimulant (mL plant^-1^)	Dry biomass (mg plant^-1^)			SPAD index	*F*_v_/*F*_m_
	Leaves	Stems	Total		
0.00	570.3 c	32.8	603.1 b	50.1	0.82
0.06	569.2 c	37.1	606.3 b	53.8	0.83
0.12	787.0 a	31.6	814.6 a	51.2	0.82
0.24	700.7 ab	28.9	729.6 ab	52.3	0.82
0.48	657.0 bc	33.9	690.9 bc	48.7	0.82
Significance	Q^∗∗^	ns	Q^∗∗^	ns	ns
*R*^2^	0.53	–	0.53	–	–

*Q, quadratic; NS, ^∗∗^Non-significant or significant at *P* ≤ 0.01, respectively. *R*^2^ = coefficient of determination for quadratic regression model. Different letters within each column indicate significant differences according to Duncan’s multiple range test (*P* = 0.05)*.

**FIGURE 1:**
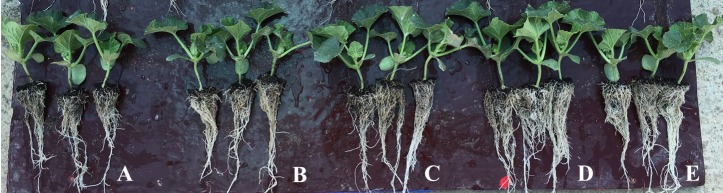
Twelve-day-old plants of melon treated with 0 **(A)**, 0.06 **(B)**, 0.12 **(C)**, 0.24 **(D)**, or 0.48 **(E)** mL plant^-1^ of biopolymer-based biostimulant.

The growth parameters in particular the dry weight of leaves as well as the total dry biomass were influenced by the vegetal-based biopolymer applications (**Table 1**). For instance, increasing the concentration of the biopolymer-based biostimulant from 0.0 to 0.48 mL plant^-1^ incurred a quadratic behavior of both leaves and total shoot biomass (**Figure 1** and **Table 1**). Our experimental data indicated that biopolymer-based biostimulant at 0.12 and 0.24 mL plant^-1^ enhanced dry weight of melon leaves and total biomass by 30.5 and 27.7%, respectively, compared to biopolymer applications at 0.06 mL plant^-1^ and untreated plants, with no significant difference found between 0.12 and 0.24 mL plant^-1^ rates (**Table 1**).

### Root Morphological Characteristics

The root-to-shoot ratio as well as the average root diameter were not affected by the vegetal-based biopolymer applications. The mean root-to-shoot ratio and root diameter of melon plants recorded in the current study were 0.25 and 0.36 mm, respectively (**Table 2**). Similarly, to the effects on plant growth parameters (dry weight of leaves and total biomass), the root dry biomass and the root morphology characteristics (total root length and surface) in biostimulant-treated plants were significantly higher at 0.24 mL plant^-1^ and to a lesser extent at 0.12 and 0.48 mL plant^-1^, in comparison to untreated melon plants (**Table 2**). In fact, the root application of the commercial biopolymer biostimulant Quik-link at a rate of 0.24 mL plant^-1^ elicited significant increase in 12-days melon transplants root biomass, total root length, and surface amounting to 34.1, 32.0, and 32.6%, respectively, compared to biostimulant applications at 0.06 mL plant^-1^ and untreated plants (**Figure 1** and **Table 2**).

**Table 2:** Effect of biopolymer-based biostimulant applications on dry weight of roots, root-to-shoot ratio, total root length, average root diameter, and total root surface of melon plants grown under greenhouse conditions.

Biostimulant (mL plant^-1^)	Root dry biomass (mg plant^-1^)	Root-to-shoot ratio	Total root length (cm plant^-1^)	Root diameter (mm)	Total root surface (cm^2^ plant^-1^)
0.00	128.3 c	0.22	709.5 c	0.35	86.9 c
0.06	161.7bc	0.26	882.1bc	0.36	106.3bc
0.12	185.6 ab	0.23	1006.0 ab	0.37	121.5 ab
0.24	194.4 a	0.27	1050.4 a	0.36	128.1 a
0.48	180.3 ab	0.26	979.6 ab	0.36	120.3 ab
Significance	Q^∗∗^	ns	Q^∗∗^	ns	Q^∗^
*R*^2^	0.95	–	0.95	–	0.96

*Q, quadratic; NS, ^∗^, ^∗∗^Non-significant or significant at *P* ≤ 0.05 and *P* ≤ 0.01, respectively. *R^2^* = coefficient of determination for quadratic regression model. Different letters within each column indicate significant differences according to Duncan’s multiple range test (*P* = 0.05)*.

### Metabolic Profiling of Melon Leaves and Roots

**FIGURE 2:**
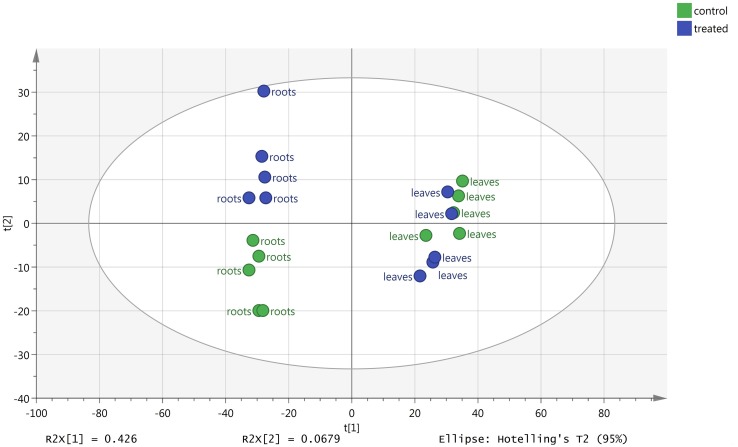
Principal Component Analysis (PCA)-based unsupervised analysis of melon roots and leaves metabolomic profile following root application of 0 (control) or 0.24 mL plant^-1^ of the biopolymer-based biostimulant.

**FIGURE 3:**
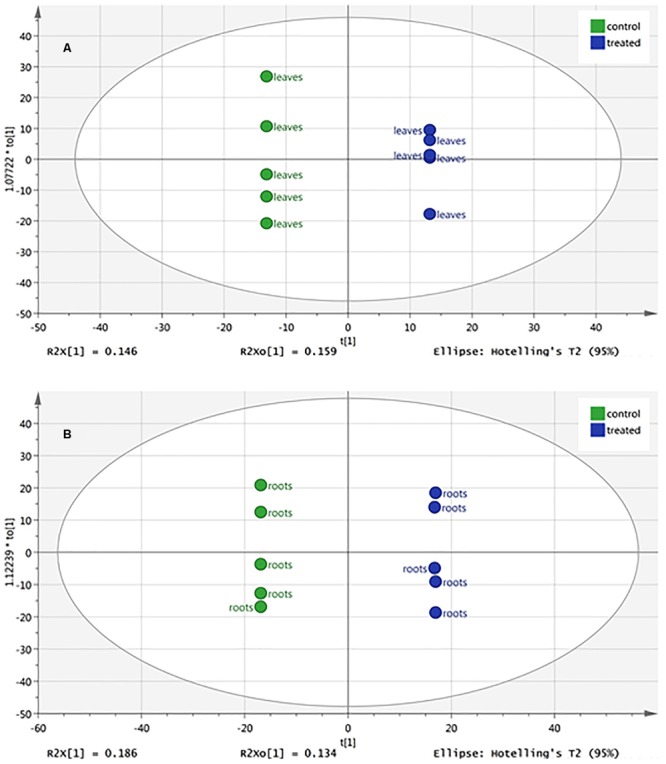
Orthogonal Projections to Latent Structures Discriminant Analysis (OPLS-DA) of melon leaf **(A)** and root **(B)** metabolomic profile following root application of 0 (control) or 0.24 mL plant^-1^ of the biopolymer-based biostimulant. Ellipses denote Hotelling’s T2 (95%).

The changes in metabolic profile of roots and leaves of melon, following treatment with the biostimulant Quik-link at the rate of 0.24 mL plant^-1^, was investigated through UHPLC-ESI/QTOF-MS untargeted metabolomics. Overall, more than 2,300 compounds were annotated and passed the filter thresholds adopted. The following unsupervised Principal Component Analysis carried out from metabolomic profiles allowed to identify two main clusters, one per matrix (**Figure 2**). Even if matrix type was, as expected, the main classification factor, two clearly distinct sub-clusters could be evidenced for root profiles, whereas leaf profiles were partially overlapped. This indicated that the effect of Quik-link was more evident in roots, and suggested the need of more focused multivariate analyses to better point out those compounds being responsible of differences across treatments. The supervised OPLS-DA model, carried out separately for roots and leaves (**Figure 3**), provided with an excellent separation of control and Quik-link treated samples. In more detail, goodness-of-fit R^2^Y was 0.62 and 0.65 and prediction ability Q^2^Y was 0.68 and 0.66, for leaves and roots respectively. Therefore, prediction ability was above the acceptability threshold of 0.5 (Rombouts et al., 2017) and was considered as acceptable. Coherently, cross-validation ANOVA resulted in in a Fischer’s probability of 0.004 for both OPLS-DA models. Furthermore, overfitting was excluded by permutation testing and no outliers could be pointed out by Hotelling’s T2 (**Supplementary Material**). Provided that differences between treatments were represented in the dataset, Volcano analysis (*P* < 0.05, fold-change > 5) allowed exporting differential compounds for roots and leaves separately (**Tables 3** and **4**, respectively). These compounds were ascribed into classes by taking into count their biochemical function and physiological roles.

**Table 3:** Differential metabolites changing in melon root after applying the biopolymer-based biostimulant, as identified through Volcano Plot analysis (*P* < 0.05, Bonferroni multiple testing correction, and fold-change cut-off = 5).

Class		Metabolite annotated	*P*-value	Fold-change	Regulation
Hormone activity	Cytokinin	Kinetin-9-*N*-glucoside/kinetin-7-*N*-glucoside	0	16	Down
		N6-(Delta2-isopentenyl)-adenosine 5′-monophosphate	0	16	Down
	Brassinosteroid	Typhasterol	0	79	Down
		6alpha-hydroxycampestanol	1.04E-13	1.89E+06	Up
		Teasterone/campesterol	0.44	8.27E+04	Up
	Auxin	Indole-3-acetyl-glutamate	0.38	9.46E+03	Down
	Gibberellin	Gibberellin A9	0	2.62E+02	Up
Stress response	Flavonoid	Quercetagetin-7-*O*-glucoside	0	1.61E+02	Up
		A 4′-hydroxyflavanone	0	16	Down
	Carotenoid	Canthaxanthin	0	16	Down
		15,15′-dihydroxy-beta-carotene	2.96E-10	9.87E+04	Up
		Zeaxanthin	0	28	Down
	Glucosinolate	2 / 3-(5′-methylthio)pentylmalate	0	16	Up
		5-methylthiopentylhydroximoyl-cysteinylglycine	0	1.21E+05	Down
		7-methylthioheptylhydroximoyl-glutathione	0	16	Down
		8-methylthiooctyl glucosinolate	0	5.96E+04	Down
Lipids	Membrane lipid	sphingosine 1-phosphate	4.81E-10	5.26E+05	Down
		1-18:1-2-16:2-monogalactosyldiacylglycerol	0	16	Up
		1-18:3-2-18:1-phosphatidylcholine	0	16	Down
Others		Salicyl alcohol	0.56	2.50E+02	Up
		4alpha-formyl-4beta-methyl-5alpha-cholesta-8,24-dien-3beta-ol	0	16	Up
		D-sedoheptulose -1,7-bisphosphate	0	6.88E+02	Down

*According to Mass Profiler Professional, P-values of 0 denote very high significance, whereas fold-change = 16 identify very high fold-changes*.

**Table 4:** Differential metabolites changing in melon leaves after applying the biopolymer-based biostimulant, as identified through Volcano Plot analysis (*P* < 0.05, Bonferroni multiple testing correction, and fold-change cut-off = 5).

Class		Metabolite annotated	*P*-value	Fold-change	Regulation
Hormones	Abscisic acid	Abscisic aldehyde	0	16	Up
		Beta-D-glucopyranosyl abscisate	0.02	3.09E+02	Down
		Antheraxanthin	0.03	3.11E+02	Up
		Zeinoxanthin	0.03	0.98E+02	Up
		3,4,3′,4′-tetradehydroisozeaxanthin	0	16	Up
	Auxin	Phenylacetate	0.04	1.02E+02	Up
		Indole acetaldehyde	0.05	6.78E+02	Down
		Indole-3-acetyl-leucine	0.05	1.08E+03	Up
	Brassinosteroid	Campesterol	0.05	0.37E+02	Down
		6-alpha-hydroxy-castasterone	0.05	1.42E+02	Up
		cathasterone	0.05	4.15E+02	Up
		(22-alpha)-hydroxy-campest-4-en-3-one	0.05	4.34E+03	Up
	Cytokinin	CPPU	0.03	0.16E+02	Up
		N6-dimethylallyladenine	0.05	0.25E+02	Up
		Zeatin-7-*N*-glucoside	0	16	Up
	Ethylene	1-aminocyclopropane-1-carboxylate	0.05	1.99E+02	Down
	Gibberellin	Gibberellin A25	0.05	1.96E+02	Down
		Ent-kaurene	0.03	0.10E+02	Up
		Ent-7-alpha-Hydroxykaurenoate	0.03	0.15E+02	Up
		Gibberellin A9 methyl ester / gibberellin A12	0.05	1.55E+03	Up
	Jasmonate	(S)-13-Hydroperoxylinolenate	0.05	0.41E+02	Down
Stress response	Oxidative stress	3-hexenal	0	16	Up
		Hydroxycaprate	0	16	Down
	Alkaloid	(S)-corytuberine	0	16	Up
		N-formyldemecolcine	0	16	Down
	Carotenoid	Bixin	1.14E-07	7.65E+04	Down
		3,5-dihydroxy-6,7-didehydro-5,6-dihydro-12’-apo-beta-caroten-12’-al	0	16	Up
		4-methylocta-2,4,6-trienedial	0	16	Up
		3-hydroxy-beta-ionone	0	16	Up
		Zeaxanthin	0.05	1.11E+04	Up
	Flavonoid	Sakuranin	2.22E-12	9.86E+04	Up
		6a-hydroxymaackiain	0	16	Up
		2,5,7-trihydroxy-4’-methoxyisoflavanone	0	16	Up
		Delphinidin-3-*O*-beta-D-glucoside	0	16	Down
		Cyanidin-3-*O*-rutinoside-5-*O*-beta-D-glucoside	0	16	Up
		2′,3,4,4′,6′-pentahydroxychalcone 4′-*O*-beta-D-glucoside	0	16	Up
Lipids	Membrane lipid	1-18:3-2-16:0-monogalactosyldiacylglycerol	0	16	Up
		Acetylcholine	1.95E-11	1.48E+05	Up
	Sterol	4-alpha-carboxy-4-beta-methyl-5-alpha-cholesta-8-en-3-beta-ol	0	16	Up
		Stigmasterol	0	16	Up
		Delta24-25-sitosterol	0	16	Down
		11-alpha-hydroxy-9,15-dioxoprost-13-enoate	0	16	Up
Others		5-deoxystrigol	0	2.58E+04	Down
		18-episcalar-17(25)-en-19-ol	0	16	Up
		2-methoxy-6-*all trans*-octaprenyl-2-methoxy-1,4-benzoquinol	0	16	Up
		Primary fluorescent chlorophyll catabolite	0	16	Up
		Delta1-pyrroline-2-carboxylate	0	16	Up
		4-(1-methyl-2-pyrrolidinyl)-3-oxobutanoate methyl ester	0	16	Up
		4-methylthiobutylhydroximoyl-cysteinylglycine	0	16	Down

*According to Mass Profiler Professional, P-values of 0 denote very high significance, whereas fold-change = 16 identify very high fold-changes*.

Regarding roots, most of the compounds were related to stress response (i.e., flavonoids, carotenoids, and glucosinolates) rather than hormone network (mainly brassinosteroids and cytokinins), followed by membrane lipids and other compounds. Although a well-defined trend cannot be observed for membrane lipids, flavonoids and carotenoids, glucosinolates were down-accumulated (excepting for 2/3-(5′-methylthio)pentylmalate). Cytokinins were also down-accumulated whereas brassinosteroids were generally up-accumulated (**Table 3**). VIP analysis from OPLS-DA supervised modeling (**Supplementary Material**) confirmed the involvement of brassinosteroids, being 6-alpha-hydroxycampestanol (isobaric with 3-epi-6-deoxocathasterone and 6-deoxocathasterone), campest-4-en-3beta-ol and campesterol among the compounds with the highest VIP score. Notably, other top-ranking OPLS-DA VIP score compounds such as the carotenoids zeaxanthin and 15,15′-dihydroxy-beta-carotene, were in common with Volcano plot analysis.

However, Volcano Plot analysis highlighted that the changes in leaves metabolic profile following application of Quik-link was distinct from response in roots (**Table 4**). A complex and wide alteration of hormone profile, involving among other brassinosteroids, auxin, abscisic acid (ABA), cytokinins, and gibberellins, was observed. The most clear trends were related to the increase of ABA intermediates (excepting the ABA glucose ester), brassinosteroids (excepting the precursor campesterol), and cytokinins. Furthermore, several stress-related compounds were selected by Volcano Plot, namely the oxidative stress-related 3-hexenal and hydroxycaprate, few alkaloids, carotenoids (all of them up-accumulated excepting for bixin) and flavonoids (also found to be up-accumulated). Membrane lipids and sterols were also altered by the treatment, together with compounds from other classes. Among these latter, pyrrole-related (delta1-pyrroline-2-carboxylate and 4-(1-methyl-2-pyrrolidinyl)-3-oxobutanoate methyl ester) and photosynthesis-related (2-methoxy-6-*all trans*-octaprenyl-2-methoxy-1,4-benzoquinol and a primary fluorescent chlorophyll catabolite) metabolites were pointed out. Discriminating compounds gained by VIP analysis following OPLS-DA confirmed the involvement of brassinosteroids (26-hydroxybrassinolide), membrane lipids (a lysophosphatidylcholine and a digalactosylglycerol), and photosynthesis-related compounds (haematoporphyrin IX, menaquinol-8, and dimethyl phylloquinone) in response to Quik-link treatment (**Supplementary Material**).

## Discussion

The use of biopolymer-based biostimulant in vegetable cropping systems can promote root growth leading to a better transplant establishment, and higher crop productivity. The current study demonstrated that substrate drench of a biopolymer-based biostimulant elicit dose-dependent (especially at 0.12 and 0.24 mL plant^-1^) increases of biomass production of melon transplants. A presumed mechanism behind the stimulation of biomass production in response to root application of vegetal-based biopolymers could be the presence of *signaling compounds* in particular bioactive peptides (e.g., LRPP) as well as lignosulfonates. The former *signaling molecules* in the Quik-link formulation which is easily perceived by root organ may have triggered a signal transduction pathway through modulation of various endogenous phytohormone biosynthesis (Matsumiya and Kubo, 2011; Colla et al., 2017a). Our findings are in agreement with the results of several research teams (Ertani et al., 2011; Matsumiya and Kubo, 2011; Colla et al., 2013, 2014) who showed that foliar of root applications of small peptides and lignosulfonate-humate elicited hormone-like activities on a wide range of agronomic and horticultural crops (baby’s-breath, cabbage, Italian clover, and lettuce, maize and tomato), thus boosting plant growth and yield.

Another putative mechanism behind the enhancement of plant growth parameters (dry weight of leaves and total biomass) induced by Quik-link application could be attributed to the stimulation of root morphology characteristics in particular the total root length and surface area (at 0.24 mL plant^-1^ and to a lesser extent at 0.12 and 0.48 mL plant^-1^), which may improve nutrient uptake and utilization efficiency with beneficial effect on biomass production (Matsumiya and Kubo, 2011). Our results are in line with previous studies assessing the stimulation action of a plant-derived protein hydrolysate containing bioactive peptides and commercial lignosulfonate-humate on root biomass and morphology (Ertani et al., 2011; Colla et al., 2014). For instance, Ertani et al. (2011) demonstrated that the application of lignosulfonate-humates to the nutrient solution (applied at 0.0, 0.5, or 1.0 mg L^-1^) elicited a dose-dependent increase of root dry weight (from 17 to 24%) in hydroponically-maize compared to the untreated control. Similarly, Colla et al. (2014) showed that the root application of 6 mL L^-1^ of a plant-derived protein hydrolysate increased root dry weight, length, and surface area of tomato cuttings by 24–35% in comparison to untreated plants.

Quik-link application resulted in a metabolic reprogramming, with roots undergoing a stronger change in biochemical profile. Indeed, despite Volcano Plot analysis highlighted a higher number of differential compounds in leaves, PCA unsupervised multivariate statistics showed that the metabolic profile of roots was markedly different in treated samples, as compared to control. Metabolomics was used to better understand the changes induced by the treatment and to highlight the processes underlying the morphological changes observed. Roots response to Quik-link application involved compounds falling into two main processes, namely stress response and hormone profile.

Among the latter, brassinosteroids appeared to play a pivotal role, with several compounds pointed out both by Volcano Plot analysis (most of them being up-accumulated) and by OPLS-DA VIP analysis. Brassinosteroids are polyhydroxy steroid lactone hormones that are essential for normal plant growth, having effect on various developmental processes of plants (Li and Chory, 1999; Zhu et al., 2013). They are produced from plant sterols, via teasterone, typhasterol, and castasterone, by an isoprenoid biosynthetic pathway that includes acetyl CoA (Ali, 2017). Brassinosteroids signaling pathway leaded to the identification of a putative brassinosteroid receptor as well as brassinosteroid-responsive genes (Li and Chory, 1999; Lin et al., 2017). These hormones are implicated in a wide range of physiological and biochemical responses in plants, including seed germination, cell division and elongation, vascular differentiation, photomorphogenesis, photosynthesis, and senescence (Ali, 2017). They have also been found to protect plants from both abiotic and biotic stress factors, such as salinity, drought, heavy metals, and pathogens (Ali, 2017). Interestingly, beside promoting crop yield, they are reported to regulate plant architecture (Lin et al., 2017), to promote root growth in a dose-dependent manner (Mussig, 2003) and to have an important role in directing epidermal cell fate in roots, regulating differentiation into hair or non-hair cells (Zhu et al., 2013). Therefore, the changes of brassinosteroids in plant tissues can help to explain the increased root development following Quik-link application. Indeed, these hormones display direct interaction with gibberellins signaling pathway, promoting mitotic activity and expression of cyclins in the root meristem (Zhu et al., 2013). In general, brassinosteroids are reported to have a wide interplay with several hormones including an antagonistic role toward ABA (Clouse, 2016) and ethylene biosynthesis (Zhu et al., 2015).

It is described that exogenous brassinosteroids are taken up through the roots to be translocated, unchanged, to the shoot (Symons et al., 2008). However, the same authors reported that, when applied directly to shoot tissues, exogenous brassinosteroids are relatively immobile. Coherently, brassinosteroids were found among differential compounds also in leaves both following Volcano Plot analysis (with 6-alpha-hydroxy-castasterone, cathasterone, and 22alpha-hydroxy-campest-4-en-3-one being up-accumulated) and OPLS-DA VIP analysis (26-hydroxybrassinolide). Several other hormones were found among differential compounds in leaves; the ethylene precursor 1-aminocyclopropane-1-carboxylate and the jasmonate precursor 13-hydroperoxylinoleate were both down-accumulated. Moreover, ABA precursors were up-accumulated, and its storage form beta-D-glucopyranosyl abscisate was down-accumulated. This might suggest that ABA biosynthesis was hampered by brassinosteroids. Furthermore, cytokinins and the most of gibberellins were both up-accumulated, whereas a clear trend could not be observed for auxins. Generally, brassinosteroids induced response appeared to affect the complex interplay and cross-talk between plant hormones, in agreement with previous literature (Li and Chory, 1999; Zhu et al., 2015; Clouse, 2016; Lin et al., 2017).

A correlation between net photosynthesis and brassinosteroids action has been also reported (Ali, 2017); indeed, a benzoquinol, a chlorophyll catabolite, and two pyrrole-related intermediates were up-accumulated in leaves following application of Quik-link. Similarly, hematoporphyrin IX, menaquinol-8, and demethylphylloquinone were among the compounds with the highest VIP score in OPLS-DA.

In a previous study focused on the effect of brassinosteroids on chill injury of fruits and vegetables during post-harvest, Soleimani Aghdam et al. (2016) reported: (i) enhanced membrane integrity, (ii) enhancing antioxidant capacity, and (iii) alteration in phenylalanine ammonia-lyase (PAL) and polyphenol oxidase (i.e., two key enzymes in phenolics biosynthesis and degradation) activities. Similarly, carotenoids were induced in tomato fruits following application of brassinosteroids (Zhu et al., 2015). This might explain the involvement of flavonoids and carotenoids in roots and, more markedly, in leaves (where both were up-accumulated). Nonetheless, these responses related to flavonoids and carotenoids, together with the changes in alkaloids and glucosinolates (two additional classes of metabolites displaying a pivotal role in stress response) are likely connected, at least in part, into the brassinosteroids-dependent response to biotic and abiotic stresses.

## Conclusion

The application of the commercial vegetal-based biopolymer Quik-link as substrate drench improved the melon growth parameters in a dose-dependent manner. The improve in plant biomass production was associated to the stimulation of the root growth and morphology traits, thus inducing a *‘nutrient acquisition response’* that favors nutrient uptake and utilization efficiency. Metabolomics allowed to point out a wide and complex biochemical response to the treatment, in which hormone profile, and brassinosteroids in particular, appeared to play a pivotal role. Root metabolic profile was markedly altered following application of the test product. In roots, brassinosteroids could have been responsible of changes in root development. These hormones are known to be transported to shoots, where they might have interfered with other hormones through a complex and wide cross talk that resulted in the hormonal imbalance. Besides potentially shaping plant structure, brassinosteroids were likely responsible of the changes in other secondary metabolites such as phenolics and carotenoids, as well as the modulation of photosynthetic activity. Regardless the eventual direct role of brassinosteroids, the treatment triggered an up-accumulation of several metabolites involved in defense mechanisms against biotic and abiotic stresses, such as flavonoids, carotenoids, and glucosinolates. Although more focused and dedicated experiments are needed in this direction, this could result in an improved resistance toward plant stress.

## Author Contributions

LL performed the metabolomic study, analyzed the data, and wrote many parts of the manuscript. YR performed the agronomic study, analyzed the data, and wrote many parts of the manuscript. MC performed the root growth measurements and implemented the manuscript. PB gave support in the data analysis and interpretation. CB collaborated in the metabolomic study. GC provided intellectual inputs for defining the experimental design, data analysis and interpretation, and improved the manuscript.

## Conflict of Interest Statement

The authors declare that the research was conducted in the absence of any commercial or financial relationships that could be construed as a potential conflict of interest.

## Footnotes

1 http://www.plantcyc.org

2 http://cosmos-fp7.eu/msi

## Supplementary Material

FIGURE S1 Hotelling’s T2 investigation on outliers as carried out in UHPLC/QTOF-MS metabolomic investigations on root and leaf metabolic profile following root application of the biopolymer-based biostimulant.

FIGURE S2 Visual representation of the VIP-selected discriminating compounds from the OPLS-DA modeling on roots **(A)** and leaves **(B)** metabolic profile, following root application of the biopolymer-based biostimulant.

TABLE S1 VIP-selected discriminating compounds from OPLS-DA modeling on root and leaf metabolic profile, following root application of the biopolymer-based biostimulant.

TABLE S2 Whole dataset gained from UHPLC/QTOF-MS metabolomic investigations on melon metabolic profile following root application of the biopolymer-based biostimulant. Compounds abundance, together with retention time and composite spectra (mass-abundance pairwise) are provided.
