# Supplementary figures and images for: A Vegetal Biopolymer-Based Biostimulant Promoted Root Growth in Melon While Triggering Brassinosteroids and Stress-Related Compounds
Source: Front Plant Sci. 2018 Apr 10;9:472. doi: 10.3389/fpls.2018.00472 (PMC5902679; doi:10.3389/fpls.2018.00472)

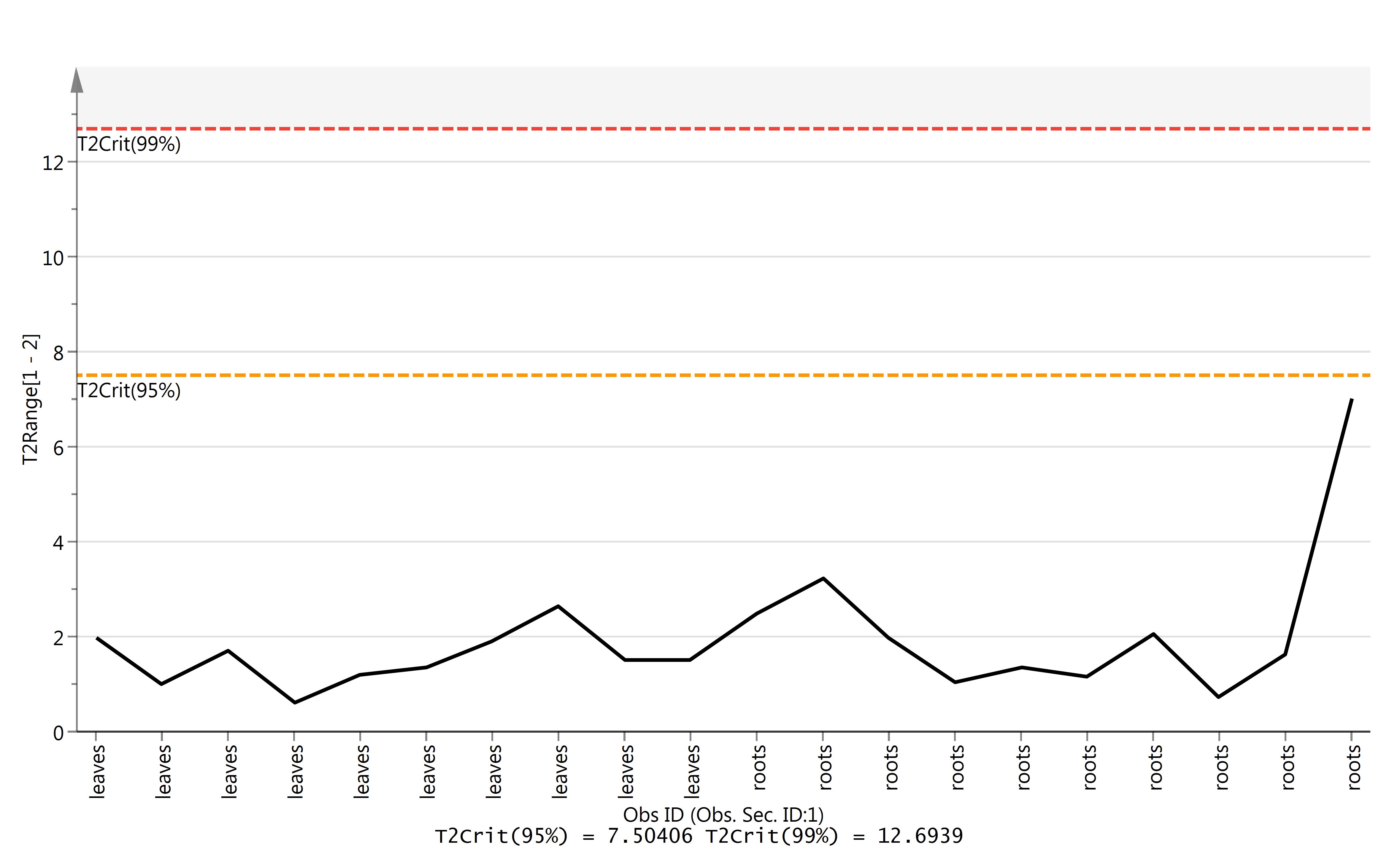

Supplement: FIGURE S1 — Hotelling’s T2 investigation on outliers as carried out in UHPLC/QTOF-MS metabolomic investigations on root and leaf metabolic profile following root application of the biopolymer-based biostimulant. [file Image_1.JPEG]

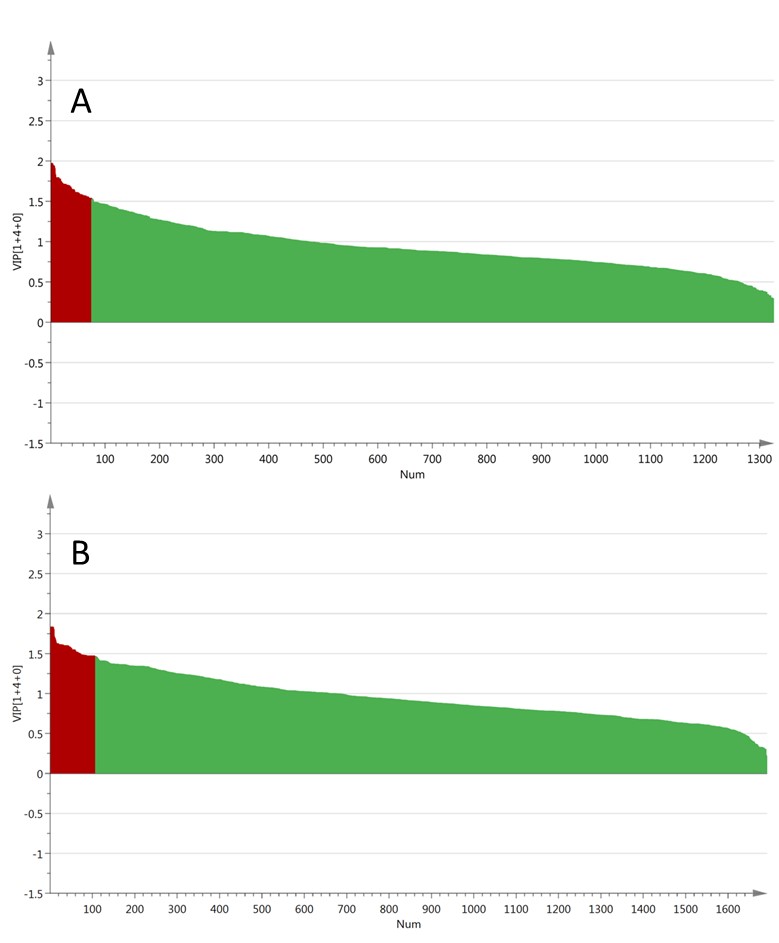

Supplement: FIGURE S2 — Visual representation of the VIP-selected discriminating compounds from the OPLS-DA modeling on roots (A) and leaves (B) metabolic profile, following root application of the biopolymer-based biostimulant. [file Image_2.JPEG]
